# Habitat use in south-west European skinks (genus *Chalcides*)

**DOI:** 10.7717/peerj.4274

**Published:** 2018-01-12

**Authors:** Daniel Escoriza, Guillem Pascual, Alberto Sánchez-Vialas

**Affiliations:** 1 Institut Català de la Salut, Barcelona, Spain; 2 Laboratory of Ecology, University Abdelmalek Essaâdi, Tetouan, Morocco; 3 Galanthus, Celrà, Girona, Spain; 4 Museo Nacional de Ciencias Naturales (MNCN-CSIC), Madrid, Spain

**Keywords:** *Chalcides bedriagai*, *Chalcides striatus*, Iberian Peninsula, Microhabitat, Niche overlap

## Abstract

**Background:**

Congeneric species of reptiles frequently exhibit partitioning in terms of their use of habitats or trophic resources in order to reduce competition. In this study, we investigated habitat use by two species of European skinks: *Chalcides bedriagai* and *Chalcides striatus*, based on 49 records from southern France, Spain, and Portugal.

**Methods:**

We measured three levels of niche descriptors: macroscale (climate, topography, and substrate), mesoscale (plant associations), and microscale (vegetation cover and shelters). We assessed the associations between these environmental descriptors and the occurrence of the skinks.

**Results:**

Our results showed that the two species occupied opposite extremes of the ecological gradient i.e., *C. bedriagai* in semi-arid environments and *C. striatus* in temperate-oceanic environments, but there was broad ecological overlap in transitional climates at all of the habitat scales examined. This overlap was demonstrated by the presence of syntopy in geographically distant sites with different environmental characteristics.

**Discussion:**

The morphological differences between the two species, and possibly their different use of microhabitats, might favor this mesoscale overlap between congeneric species, which is relatively unusual in Mediterranean lizards.

## Introduction

The organization of biotic communities is an ongoing area of research in ecology. These studies have shown that the diversity of communities is determined by the environment (e.g., by primary productivity or habitat complexity; Koh, Lee & Lin, 2006) and by the interactions established among co-occurring species (Petchey & Gaston, 2002). Phylogenetically related species share functional traits and tend to use similar resources (e.g., basking spots and prey), thereby becoming mutually exclusive (Stuart et al., 2014). Thus, biotic communities frequently exhibit a phylogenetic structure (Cavender-Bares, Keen & Miles, 2006).

Lizards comprise one of the most diverse reptile groups in warm temperate regions where they form rich communities in terms of species (Arnold, 1987). In these communities, congeneric species tend to be segregated, thereby reducing negative interactions via the differential use of microhabitats (Capula, Luiselli & Rugiero, 1993; Vanhooydonck, Van Damme & Aerts, 2000). Mediterranean skinks are a group of lizards characterized by semi-fossorial habits (Caputo, Lanza & Palmieri, 1995), with their communities being structured according to species morphology and the type of substrate (Attum, Eason & Cobbs, 2007).

In this study, we investigated habitat partitioning in two species of skinks that are endemic to south-western Europe i.e., Bedriaga’s Skink *Chalcides bedriagai* (Boscá, 1880) and the Western Three-toed Skink *Chalcides striatus* (Cuvier, 1829). These species occur throughout most of the Iberian Peninsula and the Mediterranean regions of southern France (Salvador, 1998; Cheylan et al., 2012). The ranges of both species overlap in a large part of this region (Pollo, 2004a; Pollo, 2004b) and they can occur in syntopy (Salvador, 1998; Cabana, 2010), thereby suggesting that they use the same habitats, at least occasionally. *Chalcides striatus* has a slender body shape and small limbs with a reduced number of digits, which facilitate its movement through dense layers of grass (Caputo, Lanza & Palmieri, 1995). *Chalcides striatus* is frequently associated with wet grasslands and it has been suggested that is a habitat specialist (Pollo, 2004a; Cheylan et al., 2012). By contrast, *C. bedriagai* exhibits generalist morphology, with robust limbs and pentadactyl feet, and occurs in a broad range of habitats (Malkmus, 2004).

We characterized the niches occupied by these skinks based on macroecological (climate, topography, and substrate) and habitat/microhabitat (plant associations, vegetation structure and shelter types) variables. Some level of niche partitioning could be expected (hypothesis i) in a similar manner to other congeneric lizards that overlap geographically and share similar resources (Arnold, 1987). However, the fact that both species occur in syntopy suggests that there may also be habitat overlap, which could be restricted by ecotonal habitats (Leache & Cole, 2007) or a lack of spatial structure (hypothesis ii).

## Materials and Methods

### Study region and surveys

**Figure 1:**
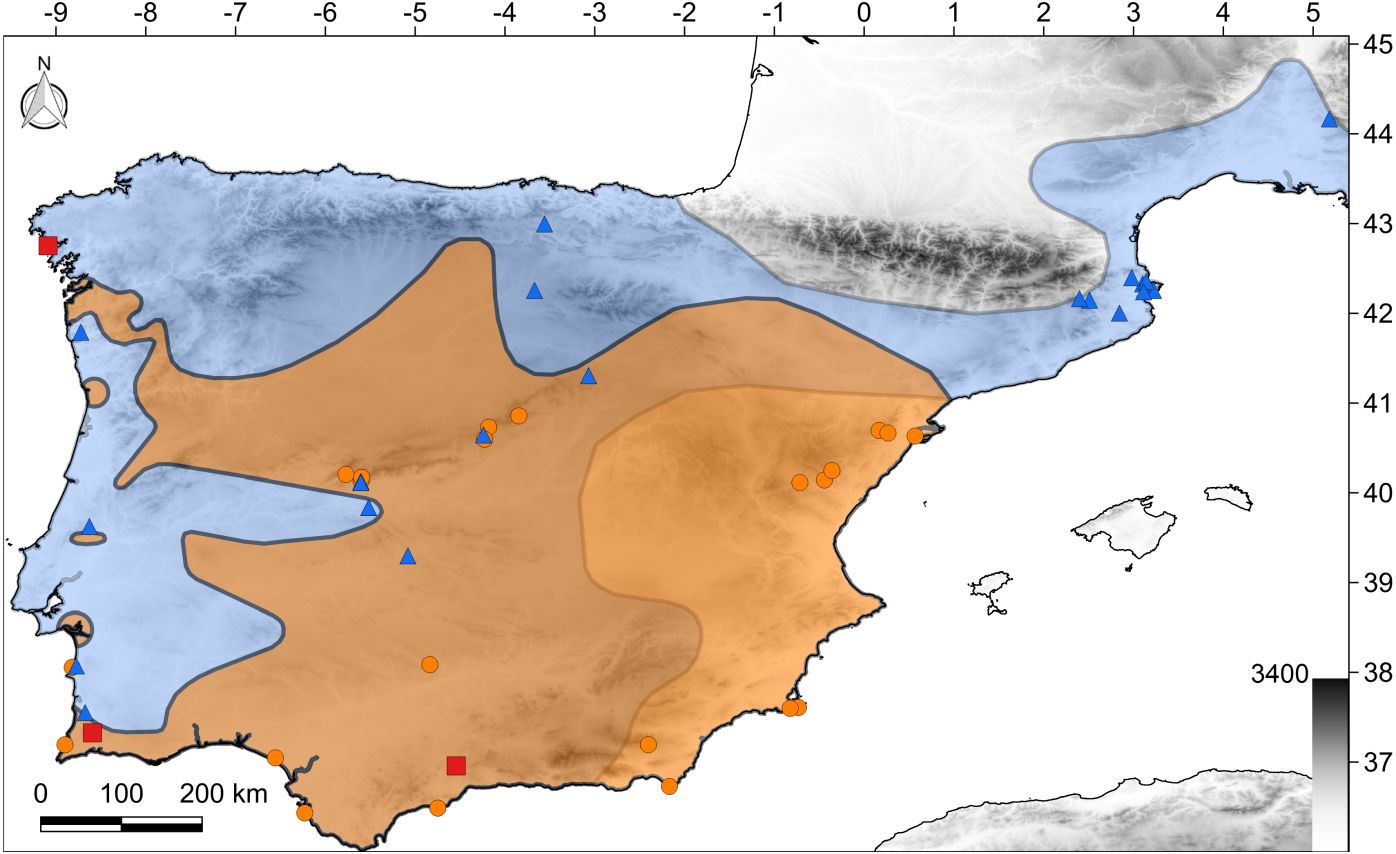
Map of the study region. The polygons show the distribution of both species according to the IUCN (2017): *C. bedriagai*, orange color; *C. striatus*, blue color; both, brown color. Orange circles, *C. bedriagai* sites; blue triangles, *C. striatus* sites; red squares, syntopy sites.

The study region was the Iberian Peninsula and south-east France, which encompass a large part of the distribution of both species (Fig. 1). Most of this region has a Mediterranean climate (types *Csa* and *Csb*; Köppen classification). However, humid/oceanic climates (types *Cfa* and *Cfb*) occur in the extreme north, semi-arid climates (types *Bsk*/*Bsh*) in the central and south-eastern parts, and desert climates (types *Bwk*/*Bwh*) on the south-eastern coast (AEMET, 2011).

We considered all possible habitat types (ranging from alpine grasslands to coastal dunes and peri-urban environments) throughout the known distributions of both species (Fig. 1). The occurrence of species was assessed based on visual surveys and rock flipping because both techniques have been used in previous studies of the presence/absence of diurnal reptiles (Losos, Marks & Schoener, 1993; McDiarmid et al., 2012). All of the sites were visited at least two times by two surveyors during the period of maximum activity for these species (early spring to early summer; Salvador, 1998). Field work was conducted over a period of 12 years as part of a study of the region’s herpetofauna (Escoriza et al., 2016). Permits for field work were provided by the Departamento de Conservação da Natureza e Florestas do Algarve and the Departament de Medi Ambient de Catalunya (ref. SF/574).

### Environmental data

At the macroscale level, we used several climate and topography variables that influence the occurrence of reptiles in temperate-subtropical regions (Anderson, 1999). We also included the substrate because it forms part of the habitat of lizards (Zaady & Bouskila, 2002; Escoriza, in press). The climate was characterized based on an aridity index (mean annual precipitation/mean annual potential evapotranspiration; Trabucco & Zomer, 2009) and the mean annual temperature (Hijmans et al., 2004). The aridity index ranges between >0.65 (humid), 0.50–0.65 (dry sub-humid), 0.20–0.50 (semi-arid), 0.03–0.20 (arid) and <0.03 (hyper arid). The climate data were obtained at a resolution of 1,000 m pixel^−1^ (Hijmans et al., 2004). The topography was characterized based on elevation, which was determined *in situ* using a global positioning system (Garmin Etrex 10; Garmin Ltd., Olathe, KS, USA). We examined the substrate texture at the first standard depth (0–5 cm) based on the sand (grain size 50–2,000 µm) and clay (grain size <2 µm) content, and the soil depth to bedrock. The soil data were obtained at a resolution of 250 m pixel^−1^ (Hengl et al., 2017). The data of these variables were extracted by the package QGIS vs 2.18 (QGIS Development Team, 2017).

We also characterized the habitats and microhabitats of the species, where we measured 37 parameters to describe the composition and structure of the habitats at a fine spatial resolution (50–0.12 m; Cerqueira & Freitas, 1999; Guénnette & Villard, 2005). Data regarding plant density and types were collected by sampling along two transects, where each measured 50 m in length and they radiated out from the center (i.e., where we found a specimen of the target species) in two opposite directions (north and south). Spermatophytes were classified according to a standard botanic classification based on growth type, stem lignification, and life cycle (Flora Iberica, 2017). The growth types were trees (species with a maximum height >5 m), bushes (1–5 m), sub-shrubs (<1 m), and lianas (climbing plants). Stem lignifications separated woody plants (trees, bushes, sub-shrubs, and some lianas), suffrutices (similar to sub-shrubs, but only lignified at the base), and grasses (plants without woody parts). The life cycle types were perennial, deciduous, and semi-deciduous for woody plants, and annual, biennial, semi-perennial, and perennial grasses (Flora Iberica, 2017). Monilophytes (ferns and equisetums) were classified as semi-perennial or perennial types (Flora Iberica, 2017).

We also assessed some characteristics of the microhabitat (at 5–0.12 m), where these variables described the density of vegetation cover and the number and type of potential shelters (Cerqueira & Freitas, 1999). We measured the distance to a forest edge (in meters up to a maximum of 1,000 m), rock surface exposure (%), soil surface exposure (%), number of stones, number of stumps, number of trees with a diameter at breast height (DBH) greater than 0.75 m, canopy cover (%, measured at 1.5 m using a spherical crown densitometer; Forestry Suppliers, Inc., Jackson, MS, USA), woody plant and grass species and stem density, and litter depth (cm).

### Data analyses

We first visualized the multidimensional niches of species using principal component analysis (PCA). In subsequent analyses, we tested the variables separately at three spatial levels, i.e., at the macroscale (1,000–250 m), mesoscale (50 m), and microscale (5–0.12 m) levels. This hierarchical approach allowed that the number of cases per variable was always greater than two, which is the minimum required for an adequate estimation of the coefficients in linear regression analyses (Austin & Steyerberg, 2015). The associations between the predictor variables and the presence of *C. bedriagai* and *C. striatus* were modeled using distance-based linear models (DistLM), where we transformed the dependent variable into a matrix of Sørensen distances (Clarke & Gorley, 2006). This analysis was used to generate the best subset of predictor variables based on a stepwise selection procedure and Akaike’s information criterion corrected for finite sample sizes (AICc; Burnham & Anderson, 2002). To assess whether the predictors had positive or negative effects on the dependent variable, we generated XY scatter plots using trend lines (Clarke & Gorley, 2006). These analyses were performed using PRIMER-E (PRIMER-E Ltd., Plymouth, UK).

We also estimated the site-level suitability. We expected that if the species occupied a well-defined ecological space (i.e., parapatric niches), then the predictions would have low cross-classification errors (Evans & Cushman, 2009). Niche suitability was estimated using random forests, which is a classification method that was designed to avoid overfitting (Broennimann et al., 2012). This property was important for our study because of the high number of variables included in the models. We set the model parameters for classification, where the variables were sampled randomly at each split as the square root of the total number of variables included in the model and we set the number of training trees to 10,000 (Liaw & Wiener, 2002). We assessed the performance of the model using the area under the receiver operating characteristic curve (AUC). The AUC values ranged from 0.5 for models with predictive ability similar to chance to 1.0 for models with perfect predictive ability (Araújo et al., 2005). These analyses were carried out with the package ‘randomForest’ (Liaw & Wiener, 2002) and ‘ROCR’ (Sing et al., 2015) in R (R Development Core Team, 2015).

## Results

**Figure 2:**
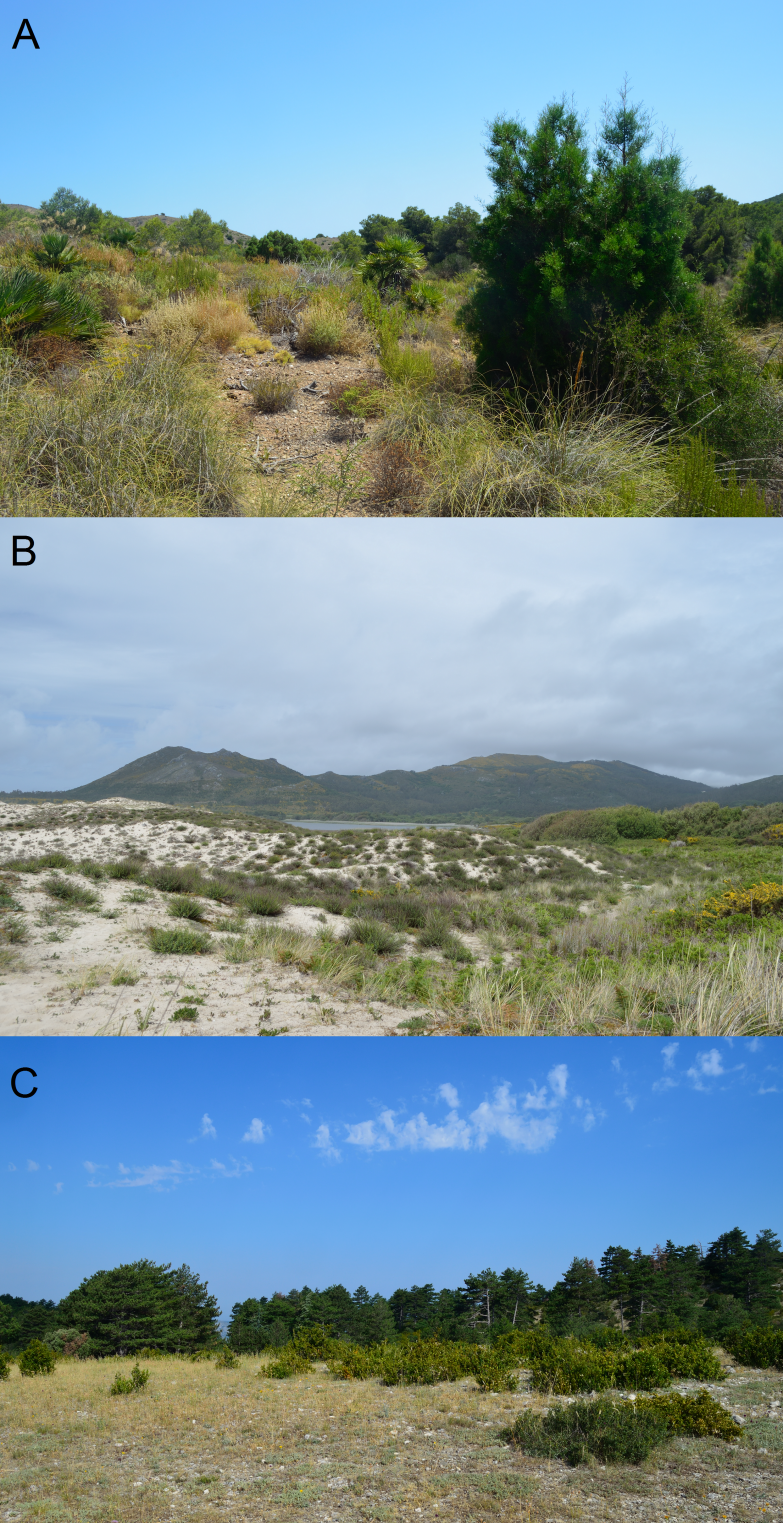
Examples of habitats occupied by the studied species. (A) habitat of *C. bedriagai*, in the arid south-east (Murcia); (B) syntopy habitat, in the coastal dunes of the north-west (Galicia); (C) habitat of *C. striatus*, in the south-east of France (Provence). Photos by Daniel Escoriza.

The target species were detected at 49 sites in Spain, Portugal, and southern France (Fig. 1). At three sites, both species were detected syntopically (Fig. 1). *Chalcides striatus* was detected using visual surveys and rock flipping whereas *C. bedriagai* was detected mostly by rock flipping. The descriptive statistics for the environmental variables are shown in Table 1. These data showed that both species overlap in a broad range of environmental parameters, occupying sparsely wooded habitats with a dense cover of grasses and shrubs (Table 1). Figure 2 shows some examples of these habitats.

**Table 1:** Descriptive (mean and range) statistics of the environmental variables. *Chalcides bedriagai*, *n* (number of sites surveyed) = 25; *Chalcides striatus*, *n* = 24.

	Scale (m)	*C. bedriagai*	*C. striatus*
Elevation (m asl)		616 (1–1,601)	476 (1–1,491)
Mean annual temperature (° C)	1,000	14.1 (7.8–18.2)	13.7 (8.6–17.1)
Aridity index	1,000	0.55 (0.24–1.34)	0.74 (0.33–1.63)
Sand texture%	250	43.0 (33.0–57.0)	41.7 (31.0–56.0)
Clay texture%	250	24.6 (16.0–32.0)	23.1 (17.0–28.0)
Soil depth (cm)	250	1,297 (431–2,053)	1,290 (337–2,112)
Needle-leaved tree%	50	0.7 (0.0–4.6)	0.1 (0.0–1.2)
Deciduous broad-leaved tree%	50	0.9 (0.0–15.1)	1.6 (0.0–6.4)
Perennial broad-leaved tree%	50	0.9 (0.0–6.6)	1.7 (0.0–5.3)
Needle-leaved bush%	50	0.5 (0.0–6.0)	0.3 (0.0–2.8)
Deciduous broad-leaved bush%	50	0.6 (0.0–6.0)	2.6 (0.0–13.9)
Perennial broad-leaved bush%	50	14.6 (0.0–58.4)	11.0 (0.0–33.0)
Subaphylla bush%	50	1.5 (0.0–24.4)	1.0 (0.0–8.8)
Sub-shrub%	50	0.7 (0.0–8.1)	0.8 (0.0–12.4)
Suffrutex%	50	22.8 (0.0–52.1)	7.8 (0.0–31.2)
Deciduous liana%	50	0.3 (0.0–2.4)	0.9 (0.0–6.8)
Semi-deciduous liana%	50	2.0 (0.0–17.2)	3.7 (0.0–17.2)
Perennial liana%	50	0.4 (0.0–6.6)	0.8 (0.0–6.4)
Perennial equisetum%	50	0.0	0.1 (0.0–2.3)
Semi-perennial fern%	50	2.0 (0.0–24.8)	2.7 (0.0–25.9)
Perennial fern%	50	0.1 (0.0–1.8)	0.4 (0.0–5.5)
Annual grass%	50	19.8 (0.0–85.1)	24.8 (0.0–79.1)
Biennial grass%	50	0.4 (0.0–3.2)	0.2 (0.0–1.5)
Semi-perennial grass%	50	1.8 (0.0–15.3)	4.2 (0.0–33.8)
Perennial grass%	50	29.7 (1.2–59.9)	34.8 (9.5–63.5)
Distance to forest edge		251 (3–1,000)	169 (7–1,000)
Rock surface exposure%	5	30 (1–70)	14 (0–40)
Soil surface exposure%	5	23 (0–75)	6 (0–30)
Number of stones	2.5	6 (0–15)	5 (0–22)
Number of stumps	2.5	0.7 (0.0–12.0)	0.1 (0.0–1.0)
Number of trees DBH > 0.75 m	2.5	0.1 (0.0–1.0)	0.2 (0.0–1.0)
Canopy cover	0.5	1.6 (0.0–20.0)	1.3 (0.0–20.0)
Wood species density	0.5	1.1 (0.0–3.0)	0.6 (0.0–2.0)
Wood stem density 0.2 m	0.5	0.7 (0.0–4.0)	0.7 (0.0–5.0)
Wood stem density 0.4 m	0.5	0.6 (0.0–9.0)	0.2 (0.0–2.0)
Wood stem density 0.6 m	0.5	0.2 (0.0–2.0)	0.1 (0.0–1-0)
Wood stem density 0.8 m	0.5	0.4 (0.0–6.0)	0.0
Grass species density	0.5	3.3 (1.0–7.0)	3.7 (2.0–6.0)
Grass stem density 0.2 m	0.5	15.8 (2.0–60.0)	36.3 (10.0–65.0)
Grass stem density 0.4 m	0.5	0.5 (0.0–10.0)	1.0 (0.0–7.0)
Grass stem density 0.6 m	0.5	0.2 (0.0–5.0)	1.8 (0.0–20.0)
Grass stem density 0.8 m	0.5	0.2 (0.0–2.0)	0.04 (0.0–1.0)
Litter depth (cm)	0.1	0.2 (0.0–1.0)	0.1 (0.0–1.0)

**Figure 3:**
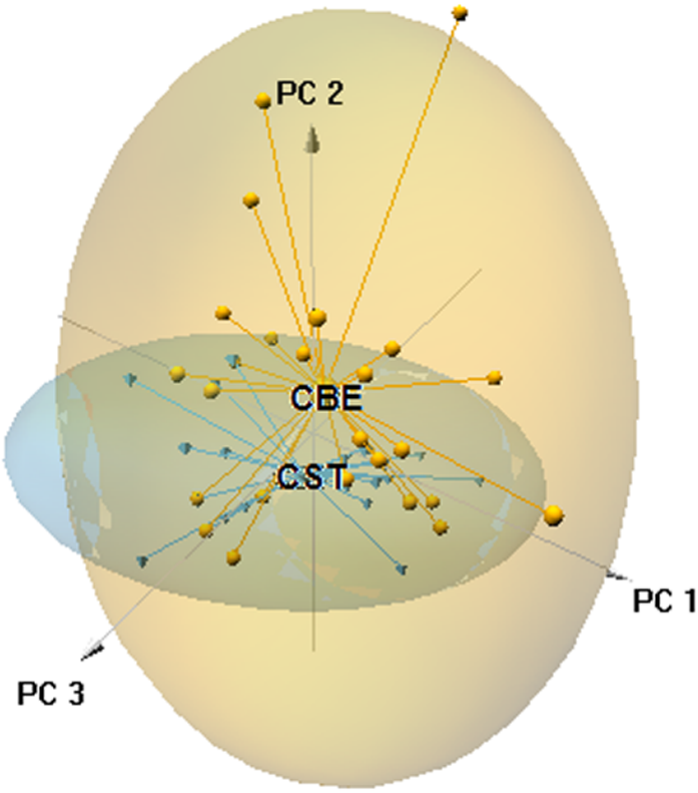
Principal component analysis scatter-plot, representing the multidimensional niche occupied by both skinks. The ellipsoids represented the 95% confidence intervals (CBE, *C. bedriagai*; CST, *C. striatus*). Circles, *C. bedriagai* sites; triangles, *C. striatus* sites. PC1 = explained variance 11.16%, PC2 = 9.06%, PC3 = 7.83%.

The ordination plot obtained by PCA based on the first three axes (explained variance = 28.05%) showed that a large part of the 0.95 confidence ellipsoid generated for *C. striatus* was included within that generated for *C. bedriagai* (Fig. 3). The DistLM analysis showed that at the macroscale, the aridity index negatively influenced the occurrence of *C. bedriagai* (Table 2), where it was the most important variable for explaining the differences between the two skinks (explained variance, EV = 10.6%; Table 3). At the mesoscale level, the proportion of suffrutex and needle-leaved trees had a positive relationship with the presence of *C. bedriagai* whereas the proportion of deciduous broad-leaved bushes had a negative relationship (Table 2). The best model at the mesoscale level included the proportion of suffrutices (EV = 26.2%) and deciduous broad-leaved bushes (EV = 4.0%). At the microscale level, rock and soil surface exposure had a positive relationship with the presence of *C. bedriagai* whereas the grass stem density (height = 0.2 m) had a negative relationship (Table 2). The best explanatory model at the microscale level included the grass stem density (height = 0.2 m) (EV = 27.9%) and rock (EV = 8.3%) and soil surface exposure (EV = 9.6%).

**Table 2:** Tests for relationships between the species presence and environmental variables, using distance based linear models.

Scale	Variables	±	Pseudo-*F*	*P*	Prop.
1,000–250 m	Elevation	+	0.97	0.327	0.021
	Mean annual temperature	+	0.20	0.645	0.004
	**Aridity index**	−	5.56	**0.021**	0.106
	Sand texture	+	0.41	0.528	0.009
	Clay texture	+	1.97	0.170	0.040
	Soil depth	+	0.04	0.953	0.001
50 m	**Needle-leaved tree%**	+	5.66	**0.018**	0.107
	Deciduous broad-leaved tree%	−	0.92	0.396	0.019
	Perennial broad-leaved tree%	−	2.05	0.163	0.042
	Needle-leaved bush%	+	0.88	0.402	0.018
	**Deciduous broad-leaved bush%**	−	6.48	0.010	0.121
	Perennial broad-leaved bush%	+	1.02	0.322	0.021
	Subaphylla bush%	+	0.20	0.836	0.004
	Sub-shrub%	−	0.05	0.863	0.001
	**Suffrutex%**	+	16.68	**0.0002**	0.262
	Deciduous liana%	−	2.68	0.102	0.054
	Semi-deciduous liana%	−	1.41	0.248	0.029
	Perennial liana%	−	0.66	0.444	0.014
	Perennial equisetum%	−	1.04	0.484	0.022
	Semi-perennial fern%	−	0.18	0.710	0.004
	Perennial fern%	−	1.41	0.292	0.029
	Annual grass%	−	0.60	0.443	0.013
	Biennial grass%	+	1.22	0.302	0.025
	Semi-perennial grass%	−	1.41	0.252	0.029
	Perennial grass%	−	1.06	0.302	0.022
5–0.12 m	Distance to forest edge	+	0.72	0.408	0.015
	**Rock surface exposure%**	+	9.70	**0.004**	0.171
	**Soil surface exposure%**	+	15.29	**0.0002**	0.246
	Number of stones	+	0.64	0.438	0.013
	Number of stumps	+	1.59	0.231	0.033
	Number of trees DBH > 0.75 m	−	0.68	0.470	0.014
	Canopy cover	+	0.07	1.000	0.002
	Wood species density	+	3.56	0.071	0.070
	Wood stem density 0.2 m	+	0.03	0.906	0.001
	Wood stem density 0.4 m	+	1.24	0.382	0.026
	Wood stem density 0.6 m	+	0.47	0.742	0.010
	Wood stem density 0.8 m	+	2.09	0.106	0.042
	Grass species density	−	1.00	0.350	0.021
	**Grass stem density 0.2 m**	−	18.22	**0.0001**	0.279
	Grass stem density 0.4 m	−	0.65	0.487	0.014
	Grass stem density 0.6 m	−	2.78	0.090	0.056
	Grass stem density 0.8 m	+	1.61	0.364	0.033
	Litter depth	+	1.34	0.416	0.028

**Notes.**

±indicates the sense (positive or negative) of the association between the species presence and the environmental predictor. The models were generated independently for each spatial scale. Significant relationships are marked in bold.

Prop. proportion of explained variance

**Table 3:** Best explanatory distance based linear models, based on a step-wise selection procedure and the Akaike information criterion corrected for finite sample sizes.

Scale	Variables	Pseudo-*F*	*P*	Prop.
1,000–250 m	Aridity index	5.56	0.021	0.106
50 m	Suffrutex%	16.68	0.0003	0.262
	Deciduous broad-leaved bush%	2.64	0.111	0.040
5–0.12 m	Grass stem density 0.2 m	18.22	0.0001	0.279
	Rock surface exposure%	5.96	0.019	0.083
	Soil surface exposure%	7.92	0.007	0.096

**Notes.**

The models were generated independently for each spatial scale.

Prop. proportion of explained variance

**Table 4:** Predicted probabilities (*C. bedriagai*= CBEŷ; *C. striatus*= CSTŷ) for the presence sites using random forest classification.

Site	Latitude	Longitude	Elevation	Observed	CBEŷ	CSTŷ
Rascafría	40.85	−3.84	1,601	CBE	0.31	0.68
Peguerinos	40.64	−4.23	1,491	CST	0.34	0.65
Gredos	40.17	−5.59	1,373	CBE	0.76	0.23
Penyagolosa	40.24	−0.35	1,301	CBE	0.66	0.33
El Espinar	40.73	−4.18	1,298	CBE	0.69	0.30
Valvenedizo	41.30	−3.07	1,235	CST	0.71	0.28
El Torcal	36.95	−4.54	1,214	Both	0.33	0.66
Robledondo	40.59	−4.22	1,174	CBE	0.43	0.56
El Boixar	40.69	0.16	1,157	CBE	0.65	0.34
Venta del Aire	40.11	−0.71	914	CBE	0.83	0.16
Velefique	37.19	−2.40	907	CBE	0.48	0.51
Zucaina	40.14	−0.44	906	CBE	0.28	0.71
Saldaña de Burgos	42.25	−3.67	887	CST	0.57	0.42
La Vall d’en Bas	42.16	2.39	886	CST	0.10	0.89
Mont Ventoux	44.16	5.18	841	CST	0.60	0.39
Monchique	37.32	−8.59	777	Both	0.30	0.69
Losar de la Vera	40.13	−5.60	643	CBE	0.43	0.56
Torme	42.99	−3.56	622	CST	0.28	0.71
Obejo	38.08	−4.83	615	CBE	0.55	0.44
Jerte	40.20	−5.77	572	CBE	0.80	0.19
Olot	42.14	2.51	564	CST	0.19	0.80
Losar de la Vera	40.12	−5.60	510	CST	0.18	0.81
Losar de la Vera	40.11	−5.60	479	CST	0.24	0.75
Castilblanco	39.29	−5.08	448	CST	0.22	0.77
La Sénia	40.66	0.26	390	CBE	0.76	0.23
Valdehúncar	39.83	−5.51	369	CST	0.37	0.62
Aldeia	41.78	−8.72	275	CST	0.52	0.47
Fátima	39.62	−8.62	258	CST	0.18	0.81
Cabo de Gata	36.72	−2.16	174	CBE	0.95	0.04
Girona	42.00	2.84	174	CST	0.14	0.85
Espolla	42.39	2.97	153	CST	0.12	0.87
Sant Carles de la Ràpita	40.63	0.56	147	CBE	0.68	0.31
Atamaría	37.59	−0.82	144	CBE	0.76	0.23
Odemira	37.54	−8.67	141	CST	0.30	0.69
Vilajuïga	42.32	3.10	42	CST	0.26	0.73
Matalascañas	37.04	−6.55	36	CBE	0.50	0.49
Sines	38.05	−8.82	21	CBE	0.90	0.09
Llancà	42.35	3.14	19	CST	0.35	0.64
Cap de Creus	42.25	3.22	18	CST	0.36	0.63
Marbella	36.48	−4.74	10	CBE	0.84	0.15
Sines	38.07	−8.77	10	CST	0.39	0.60
Carrapateira	37.19	−8.90	7	CBE	0.88	0.11
Louro	42.75	−9.09	6	Both	0.31	0.68
Calblanque	37.60	−0.73	4	CBE	0.86	0.13
San Fernando	36.43	−6.23	1	CBE	0.75	0.24
Empuriabrava	42.23	3.11	1	CST	0.37	0.62

**Notes.**

CBE *C. bedriagai* CST *C. striatus* Both syntopic sites

Datum for all coordinates, WGS84. Elevation, m asl.

The performance of the random forests model was good (AUC = 0.875). The predictions for the species and sites are shown in Table 4. The model erroneously classified 26% of the *C. bedriagai* sites and 17% of the *C. striatus* sites. The false negatives (predicted probability <0.5) for *C. bedriagai* included localities with a moderate/high herbaceous density (48.1%–87.9%) but that also included ombrophilous taxa (e.g., ferns). All of the syntopic sites were false negatives for *C. bedriagai*. The false negatives for *C. striatus* were sites with low/moderate herbaceous densities (30.4%–49.9%). At seven sites, the model predicted similar probabilities for both species (Table 4).

## Discussion

In this study, we investigated the habitat use by two species of reptiles in a broad geographical framework, where we considered the spatial scales that could hierarchically structure their occurrence. Our results showed that the inclusion of macro-, meso-, and microhabitat descriptors could be useful for understanding the complex patterns of niche occupancy by lizards.

These skinks occupied a wide environmental range, where they occurred in high mountains and coastal habitats. At the macroscale level, aridity was the factor that affected species distributions most clearly. Thus, at one of the extremes of the environmental gradient, *C. bedriagai* occupied semi-desert habitats in the coastal areas of south-eastern Spain (Pollo, 2004b). At the opposite extreme, *C. striatus* occupied the margins of the temperate-oceanic climate belt in regions with high precipitation (Pollo, 2004a). However, between these extremes, our data showed that both species overlapped in a wide range of ombroclimates. Our analyses also indicated that the characteristics of the substrate did not differentiate the sites occupied by these species.

At the meso-habitat level, we found that the habitats occupied by these skinks also differed in terms of their relative plant compositions. In the habitats occupied by *C. bedriagai*, the plant associations included higher proportions of suffrutex and needle-leaved (= conifers) trees than those where *C. striatus* was found. By contrast, in the localities occupied by *C. striatus*, the plant associations included a higher proportion of deciduous shrubs. Nevertheless, these differences could only reflect clinal variations in the vegetation as a response to the aridity gradient. Suffrutex (e.g., *Helichrysum, Lavandula*, *Thymus*) and conifer (e.g., *Pinus halepensis*) type plants dominated the arid landscapes of this region (Flora Iberica, 2017). Similarly, most of the deciduous shrubs corresponded to ombrophilous genera (e.g., *Coriaria, Ligustrum, Salix, Vitex*; Flora Iberica, 2017). At the microhabitat level, the sites occupied by *C. bedriagai* were characterized by higher substrate exposure and lower herbaceous density.

## Conclusions

Overall, these results suggest that the potential overlap between the two species is not limited to a narrow ecotonal strip, which is consistent with the syntopy found in this study in geographically distant areas located at different altitudinal levels (El Torcal de Antequera, Málaga, at 1214 m; Monchique, Algarve, at 777 m; Louro, La Coruña, at 6 m). The syntopy sites were characterized by highly suitable conditions for *C. striatus* but they could be occupied opportunistically by *C. bedriagai.* However, our surveys showed that *C. striatus* could also occupy habitats with relatively low herbaceous cover where the models predicted highly suitable conditions for *C. bedriagai.* Therefore, syntopy could be frequent but it might not always be detected due to the elusiveness of these species (Pollo, 2004a; Pollo, 2004b).

The syntopy between these species could be attributable to their thermal requirements. *Chalcides bedriagai* and *C. striatus* exhibit helio- and thigmothermal regulation, and thus both must occur in open habitats. However, within the same habitat, the more specialized morphology of *C. striatus* could favor this species occupying areas with greater herbaceous cover whereas *C. bedriagai* could occupy the habitat randomly. This hypothesis was supported by our results at this spatial level. The differences in microhabitat use could reduce mutual interactions (because they predate mainly upon small arthropods; Salvador, 1998) and favor syntopy between the two species, which is relatively unusual in congeneric Mediterranean lizards (Arnold, 1987).

The spatial overlapping patterns could be also explained by the high genetic divergence between both species (25.1 Ma; Zheng & Wiens, 2016). It is suggested that complementary niches only appear between distantly related species because unrelated species are less likely to use the same type of resources (Losos et al., 2003; Srivastava et al., 2012). In this sense, there are also examples of sibling species within the genus *Chalcides* (*C. chalcides*/ *C. striatus*, estimated divergence 10.1 Ma; Zheng & Wiens, 2016) showing parapatric distribution patterns where they meet (Sindaco et al., 2006).

##  Supplemental Information

10.7717/peerj.4274/supp-1 Data S1 Data describing the environmental variables used in this study

## Additional Information and Declarations

### Field Study Permissions

The following information was supplied relating to field study approvals (i.e., approving body and any reference numbers): Permits for field work were provided by the Departamento de Conservação da Natureza e Florestas do Algarve and the Departament de Medi Ambient de Catalunya (ref. SF/574).

### Data Availability

The following information was supplied regarding data availability: The raw data has been provided as Data S1.
